# Mevalonate Metabolism in Immuno-Oncology

**DOI:** 10.3389/fimmu.2017.01714

**Published:** 2017-12-01

**Authors:** Georg Gruenbacher, Martin Thurnher

**Affiliations:** ^1^Immunotherapy Unit, Department of Urology, Medical University of Innsbruck, Innsbruck, Austria

**Keywords:** mevalonate, metabolism, transcellular, cholesterol, fatty acid oxidation, immune cells, cancer

## Abstract

Immuno-oncology not only refers to the multifaceted relationship between our immune system and a developing cancer but also includes therapeutic approaches that harness the body’s immune system to fight cancer. The recognition that metabolic reprogramming governs immunity was a key finding with important implications for immuno-oncology. In this review, we want to explore how activation and differentiation-induced metabolic reprogramming affects the mevalonate pathway for cholesterol biosynthesis in immune and cancer cells. Glycolysis-fueled mevalonate metabolism is a critical pathway in immune effector cells, which may, however, be shared by cancer stem cells, complicating the development of therapeutic strategies. Additional engagement of fatty acidy oxidation, as it occurs in regulatory immune cells as well as in certain tumor types, may influence mevalonate pathway activity. Transcellular mevalonate metabolism may play an as yet unanticipated role in the crosstalk between the various cell types and may add another level of complexity. In humans, a subset of γδ T cells is specifically adapted to perform surveillance of mevalonate pathway dysregulation. While the mevalonate pathway remains an important target in immuno-oncology, in terms of personalized medicine, it may be the type or stage of a malignant disease that determines whether mevalonate metabolism requires training or attenuation.

## Mevalonate Metabolism in Immune Cells

### Glycolysis-Driven Mevalonate Metabolism in Immune Effector Cells

**Figure 1:**
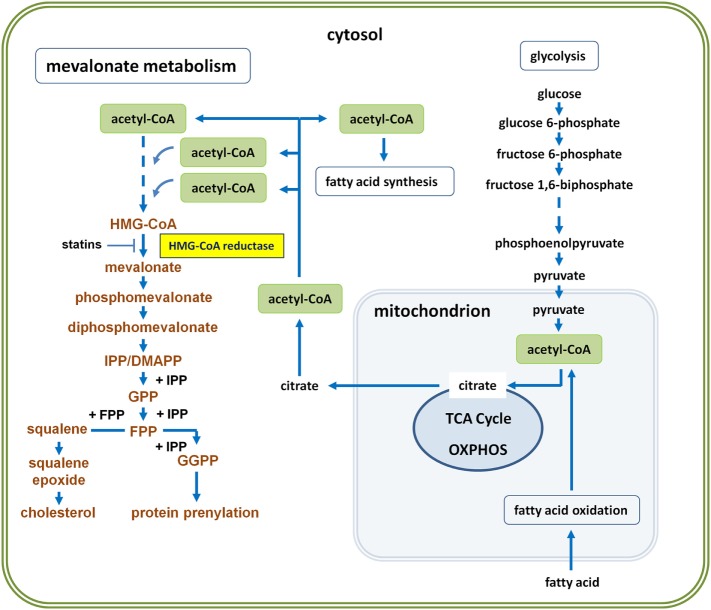
Glycolysis-driven mevalonate metabolism versus fatty acid oxidation (FAO)-driven oxidative phosphorylation (OXPHOS). Glycolysis-derived pyruvate can enter the mitochondrion and fuel the tricarboxylic acid (TCA) cycle to drive OXPHOS. Cells thus generate energy in the form of adenosine triphosphate (ATP). However, activated immune or cancer cells can also export citrate to the cytosol, where it is converted back to acetyl-coenzyme A (acetyl-CoA) by ATP citrate lyase. Abundance of cytosolic acetyl-CoA enables both, fatty acid synthesis (FAS) and mevalonate metabolism, collectively referred to as lipogenesis. Three molecules of acetyl-CoA are required to generate HMG-CoA. HMG-CoA is the substrate of HMG-CoA reductase, the mevalonate-generating enzyme, which catalyzes the first committed step and thus initiates the pathway leading to farnesyl diphosphate, also known as farnesyl pyrophosphate (FPP = branching point). Whereas FPP is the precursor in cholesterol biosynthesis, both, FPP and geranylgeranyl diphosphate (GGPP), represent activated isoprenoid moieties in posttranslational protein prenylation. Concurrent FAO may influence the availability of acetyl-CoA for mevalonate metabolism, because FAO usually serves to drive OXPHOS and thus diverts acetyl-CoA from lipogenic pathways.

Immune cell activation is associated with shifts in cellular metabolism (1, 2). In contrast to naïve T cells, T helper (Th) cells including type 1 (Th1), type 2 (Th2) as well as type 17 (Th17) display a reprogrammed metabolic phenotype, which is characterized by increased rates of aerobic glycolysis, leading to fatty acid synthesis (FAS) and mevalonate metabolism (Figure 1). Glycolysis-driven lipogenesis is induced by Akt signaling and depends on sterol regulatory element-binding protein (SREBP) transcription factors. All these changes are promoted by the metabolic checkpoint kinase mechanistic target of rapamycin (mTOR) that controls protein translation, cell growth, and metabolism (3, 4). The serine/threonine kinase mTOR exists in two complexes, mTORC1 and mTORC2, which have distinct functions. TCR triggering induces in an Akt–mTOR–SREBP-dependent manner the expression of all genes encoding mevalonate-generating and mevalonate-metabolizing enzymes (5), highlighting the importance of this metabolic pathway for T cell activation (6).

M1 macrophages, classically activated by the Th1 cytokine interferon-γ (IFN-γ) plus lipopolysaccharide (LPS), are myeloid effector cells, which are characterized by the expression of high levels of pro-inflammatory cytokines, reactive nitrogen and oxygen intermediates, promotion of Th1 response, and strong microbicidal and tumoricidal activity (7). Like Th cells, M1 macrophages also depend on glycolysis and mevalonate metabolism (2, 8). Finally, dendritic cells (DCs), which encounter bacterial components such as LPS as well as T or NK cell-derived IFN-γ during infection likewise operate glycolytic metabolism (2) and require mevalonate pathway activity for effector cytokine production (9).

M1 macrophages and DCs engage glycolysis-fueled lipogenesis to expand cellular compartments such as the endoplasmic reticulum and the Golgi as well as to prepare the entire secretory machinery for effector responses (10). For this purpose, glucose-derived cytosolic pyruvate enters the citric acid cycle, also known as the Krebs cycle or tricarboxylic acid (TCA) cycle, which takes place in the mitochondria of eukaryotic cells. However, instead of being fully oxidized in the TCA cycle, some of the pyruvate-derived mitochondrial citrate can be exported into the cytosol. ATP citrate lyase, which is phosphorylated by Akt (11), cleaves citrate and thus generates cytosolic acetyl-CoA, the precursor of FAS and mevalonate metabolism (12, 13) (Figure 1).

### Colony-Stimulating Factors Promote Mevalonate Metabolism during Myelopoiesis and M1 Macrophage Activation

Myelopoiesis is driven by colony-stimulating factors including granulocyte/macrophage-colony-stimulating factor (GM-CSF) and M-CSF, which are important for the development and function of monocytes and macrophages. In murine myelopiesis, M-CSF stimulation activated mTORC1 and mTORC1-driven glycolysis initiated a transcriptional program involving activation of the protooncogene Myc (14), which is well known to induce metabolic reprogramming, including stimulation of lipogenesis (15). Accordingly, multiple genes involved in mevalonate generation and metabolism toward cholesterol were activated in response to M-CSF treatment (14). Attenuation of cholesterol biosynthesis gene expression by deleting SREBP cleavage-activating protein impaired myelopoiesis, highlighting the crucial role of mevalonate metabolism in macrophage development.

Granulocyte/macrophage-colony-stimulating factor plays a critical role in promoting glycolysis-fueled mevalonate metabolism (8) in M1 macrophages. GM-CSF increases the glycolytic capacity of macrophages and primes them for high levels of acute glycolysis in response to LPS stimulation. LPS has long been known to promote glucose uptake in macrophages (2, 16) and this may in part be due to LPS-induced production of GM-CSF (17). GM-CSF primed macrophages not only contained higher levels of acetyl-CoA but also displayed upregulated mRNA and protein expression of HMG-CoA reductase (8), which is the target of the statins, a class of lipid-lowering drugs widely prescribed for treatment and prophylaxis of coronary heart disease. GM-CSF primed macrophages produced significantly higher levels of the pro-inflammatory cytokines TNF-α, IL-1β, IL-6, and IL-12 in response to LPS and, importantly, simvastatin prevented the GM-CSF priming effect. These observations indicated that mevalonate metabolism instructs the inflammatory response of GM-CSF primed M1 macrophages.

Although the maintenance of immature antigen-presenting DCs is facilitated by fatty acid oxidation (FAO)-driven oxidative phosphorylation (OXPHOS) in a steady state (18), maturation into cytokine-producing, immunostimulatory DCs is again driven by glycolysis (2). The increased reliance of activated DCs on glycolysis is reflected by the observation that inhibition of hexokinase by 2-deoxyglucose prevents the DC maturation process (2). A major reason for the metabolic switch of maturing DCs is the increased need for citrate, which determines the levels of cytosolic acetyl-CoA, fueling not only FAS but also mevalonate metabolism (Figure 1). In this context, it may be of interest that human monocyte-derived DCs developing in the presence of GM-CSF and IL-4 produce substantial amounts of M-CSF (19). M-CSF synthesis is rapidly induced by GM-CSF during the first 24 h of DC culture and then declines during the 5-day culture period. Given the importance of M-CSF in promoting mevalonate metabolism during myelopoiesis (14), the stimulatory effects of LPS and GM-CSF on glycolysis-fueled mevalonate metabolism may at least in part be mediated by M-CSF, which is induced by LPS and GM-CSF. However, M-CSF on its own is not capable of macrophage priming for enhanced inflammatory responses (20).

### OXPHOS Fueled by FAO in Quiescent and Regulatory Immune Cells

In contrast, other immune cell types such as naïve T cells and quiescent CD8 memory T cells, whose major task is to survive, engage OXPHOS driven by FAO (21). In addition, immune cells responsible for the limitation of inflammation to ensure the return to homeostasis such as M2 macrophages (22), which develop in the presence of M-CSF and the Th2 cytokine IL-4, as well as regulatory T (T_reg_) cells (23) and tolerogenic DCs (24) also operate FAO-driven OXPHOS (2). The role of mevalonate metabolism in these cells is less clear. Interestingly, mTORC1 signaling in T_reg_ cells has been shown to promote cholesterol and lipid metabolism. The mevalonate pathway turned out to be particularly important for coordinated T_reg_ cell proliferation and for the establishment of T_reg_ cell functional competence (25). These findings indicate that regulatory immune cells may concomitantly engage FAO and mevalonate metabolism. However, it seems possible that mitochondrial oxidation of acetyl-CoA for increased ATP synthesis may limit its availability for mevalonate metabolism. If this proves to be true, mevalonate metabolism might represent an Achilles’ heel-like target and statins may be used to enhance immunotherapy by relieving cell-mediated immunosuppression.

## Mevalonate Metabolism in Cancer Cells

Uncontrolled growth of tumors is usually promoted by aerobic glycolysis, an observation originally made by Otto Warburg almost a century ago (26). Glycolysis-driven mevalonate metabolism is potentially oncogenic, most likely *via* excessive protein prenylation (27). Physiologically, the tumor suppressor p53 controls mevalonate pathway activity; however, p53 gain-of-function mutation can lead to uncontrolled mevalonate metabolism and subsequently to malignant transformation (28). Other cancers may lack feedback control of HMG-CoA reductase (HMGCR) or overexpress of HMGCR, leading to permanently increased flux through the mevalonate pathway (29, 30). Along the same line, ectopic expression of HMGCR also facilitated malignant transformation (31).

### Myc-Driven Mevalonate Metabolism in Cancer Stem Cells

**Figure 2:**
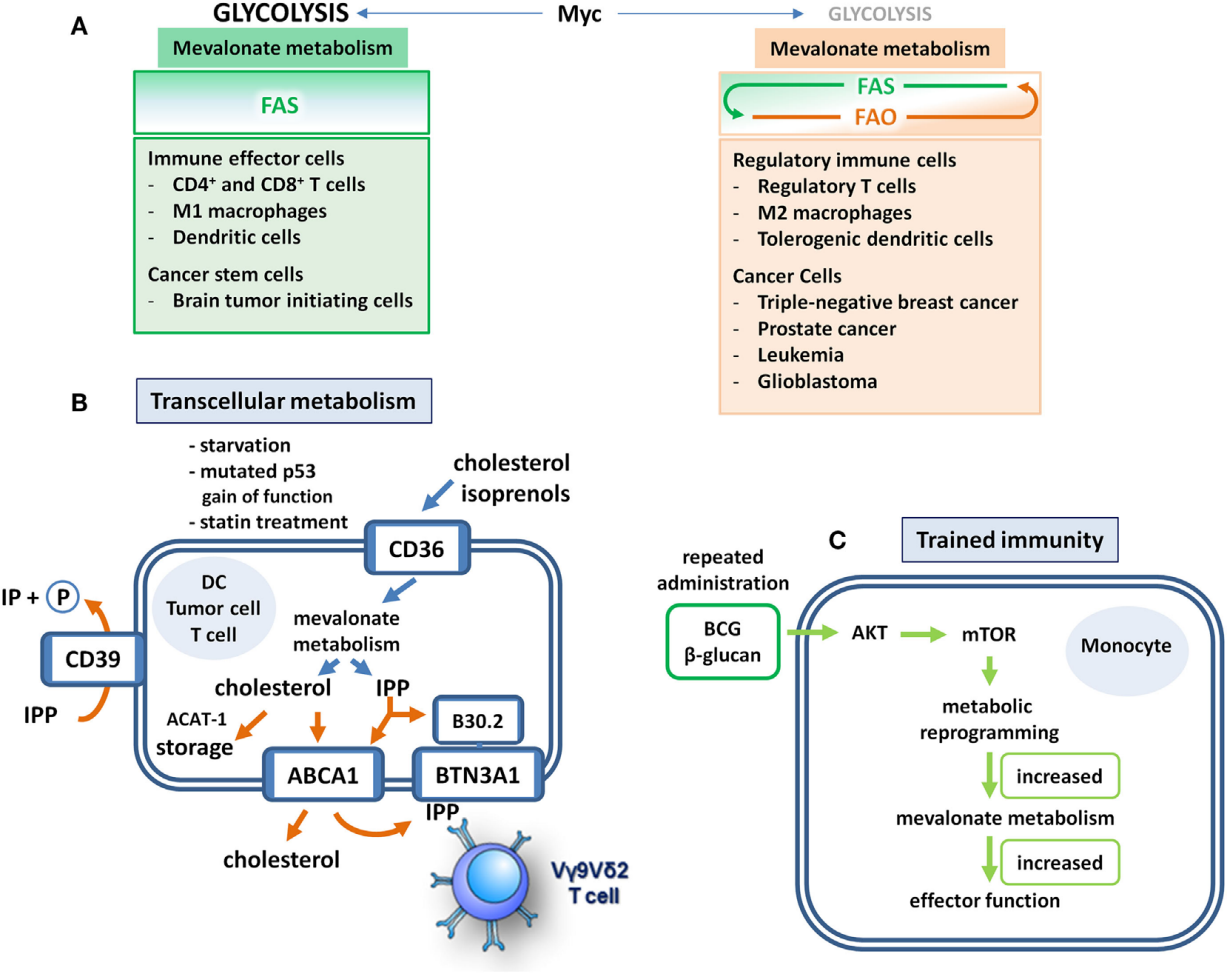
Mevalonate metabolism in immune and cancer cells. **(A)** Fatty acid metabolism makes the difference. Immune cells that realize effector functions depend on Myc-driven glycolysis that fuels lipogenesis. These cells engage mevalonate metabolism and fatty acid synthesis (FAS) but refrain from fatty acid oxidation (FAO). Unfortunately, cancer stem cells may have similar metabolic profiles. Although regulatory immune cells may still require Myc-driven glycolysis and FAS to some extent, they also engage FAO, which may occur at the expense of mevalonate pathway activity. These cells use FAO to realize their suppressive functions and to support survival. Distinct tumor types adopt a similar metabolic profile. In these cells, FAO may limit the availability of acetyl-CoA for mevalonate metabolism rendering it a potential Achilles’ heel-like target for therapeutic interventions. **(B)** Transcellular mevalonate metabolism. Starvation or p53 gain-of-function mutations lead to enforced uptake and use of extracellular isoprenoids in tumor cells. Intracellularly accumulating isopentenyl diphosphate (IPP) can bind to the B30.2 domain of butyrophilin 3A1 (BTN3A1), which leads to the activation of Vγ9Vδ2 T cells through a conformational change of the extracellular domain. In dendritic cells (DCs), the cholesterol efflux transporter adenosine triphosphate (ATP)-binding cassette transporter A1 (ABCA1) may export mevalonate-derived IPP into the extracellular space. Extracellular IPP can bind to BTN3A1 on the DC cell surface resulting in the activation of Vγ9Vδ2 T cells. The ecto-ATPase CD39 is able to dephosphorylate IPP thus limiting the duration and strength of IPP-induced γδ T cell responses. The lipid scavenger CD36 may also mediate the uptake of extracellular isoprenoids. Cholesterol storage through ACAT-1-mediated esterification may limit T cell activity. **(C)** Training of mevalonate metabolism. Priming of monocytes with Bacillus Calmette–Guérin (BCG) or β-glucan leads to an Akt–mTOR-driven metabolic reprogramming that empowers these cells to respond to subsequent challenges with increased production of cytokines and reactive oxygen intermediates. This increased responsiveness represents a form of innate memory and is based on enhanced flux through the mevalonate pathway. Significantly upregulated reactions include the production of acetoacetyl-CoA (ACAT-1), phosphomevalonate, farnesyl diphosphate as well as squalene and 2,3 oxidosqualene, the latter being rate-limiting steps of cholesterol biosynthesis. Overall, more than 50% of the genes in the pathway are activated in trained cells.

Recently, Myc has been shown to mediate its oncogenic effect by stimulating mevalonate metabolism in cancer stem cells (30) (Figure 2A), which share signaling and metabolic pathways with tumor cells upon epithelial–mesenchymal transition (32). Brain tumor-initiating cells (BTICs) were shown to exhibit enhanced mevalonate pathway activity (30). All genes encoding the enzymes that in a series of reactions convert HMG-CoA *via* mevalonate into farnesyl diphosphate (FPP) (Figure 1) were shown to be activated in BTIC models and induction of differentiation caused suppression of these mevalonate pathway genes. In addition, targeting the mevalonate pathway in BTICs by RNA interference of HMGCR expression or by pharmacological inhibition of HMGCR activity using statins attenuated proliferation, self-renewal, and tumorigenicity. Moreover, statin treatment of BTICs also reduced Myc expression.

## Transcellular Mevalonate Metabolism

An additional level of complexity has been generated by the observation of extracellular or transcellular mevalonate metabolism. This term refers to a form of short distance intercellular communication, in which lipid intermediates synthesized and released by one cell type, can be incorporated and further metabolized by another cell type. Such interaction between different cell types by shared metabolism is a well-described phenomenon during eicosanoid biosynthesis (33). Among the secretory products of activated endothelial cells is arachidonic acid, the lipid precursor that initiates the eicosanoid cascade leading to the synthesis of prostaglandins, leukotrienes, and lipoxins. Human monocytes recruited by activated endothelial cells can respond to endothelial cell-derived arachidonic acid by activating not only the eicosanoid cascade but also the *de novo* pathway of FAS. As a consequence, arachidonic acid-pulsed monocytes acquire inflammatory phenotype and function (34).

The concept of transcellular lipid metabolism also applies to the mevalonate pathway. The contribution of extracellular mevalonate to mevalonate pathway activity is usually low (5%) but may increase to >30% during periods of starvation. In addition, unusually high concentrations of extracellular isoprenols (>10 μM) may result in a relative contribution of up to 50% (35). Moreover, tumor cells carrying mutated p53, which enhances mevalonate metabolism instead of suppressing it, increasingly use extracellular isoprenols resulting in a relative contribution of even greater than 50%. Finally, treatment with statins, which blunt mevalonate metabolism by inhibiting HMG-CoA reductase, increased the use of extracellular isoprenols (35) (Figure 2B).

Isopentenyl diphosphate (IPP) can also be exported to the extracellular space. In DCs, the cholesterol efflux transporter ATP-binding cassette transporter A1 (ABCA1) has recently been shown to also mediate the efflux of IPP (36). Since cholesterol efflux serves homeostatic purposes, ABCA1-mediated IPP export will only occur in cells with high flux through the mevalonate pathway. Extracellular IPP may thus function as an indicator of hyperactive mevalonate metabolism that alerts the immune system. IPP efflux to lipid-free apolipoprotein A-I (apoA-I) results in binding of extracellular IPP to butyrophilin 3A1 (BTN3A1) on the cell surface (37, 38). BTN3A1-mediated presentation of IPP subsequently activates Vγ9Vδ2 T cells, which are innate-like T cells with considerable antimicrobial and antitumor potential (39). BTN3A1 has been shown to also serve as a sensor of intracellular IPP levels. Upon binding of IPP to its cytoplasmic B30.2 domain, conformational changes of the BTN3A1 extracellular domain facilitate TCR engagement and Vγ9Vδ2 T cell activation (40). Vγ9Vδ2 T cells activated by either pathway can then kill cells with a hyperactive mevalonate metabolism and thus contribute to the surveillance of infection or oncogenic transformation (Figure 2B).

Consistent with a role of extracellular IPP in immune surveillance, the ecto-ATPase CD39 has recently been shown to also dephosphorylate and inactivate IPP and other mevalonate-derived phosphoantigens, thus limiting the duration and strength of phosphoantigen-induced γδ T cell responses (41). Additional evidence for transcellular mevalonate metabolism has been provided by a recent study demonstrating that the lipid scavenger receptor CD36 can also mediate the uptake of extracellular isoprenoids (42). The earlier observation that statin treatment resulted in the upregulation of CD36 had already pointed toward a role of CD36 as an isoprenoid scavenger receptor (43) (Figure 2B).

Along the same line, add-back experiments, in which mevalonate metabolism of immune (44–46) and cancer cells (35, 47) could be restored by exogenous isoprenoids during drug-induced pathway inhibition, further confirmed the relevance of transcellular mevalonate metabolism. In such experiments, addition of FPP, or more often of geranylgeranyl diphosphate, was able to reinstate protein prenylation during statin or N-BP-mediated inhibition of mevalonate metabolism (48). An intriguing aspect of transcellular mevalonate metabolism is the possibility of crosstalk not only between immune cell subsets but also between immune cells, stromal cells, and cancer cells (49). The outcome of such shared metabolism is currently unclear and certainly deserves reinforced examination.

## Therapeutic Targeting of Mevalonate Metabolism

### Training of Metabolic Skills

As outlined above, mevalonate metabolism is crucial for the inflammatory response of M1 macrophages (2, 8). Intriguingly, mevalonate metabolism can apparently be trained for enhanced innate immune responses, for instance by repetitive administration of Bacillus Calmette–Guérin (BCG) (Figure 2C). Live attenuated BCG mycobacteria have a long history as a tuberculosis vaccine and as a cancer therapeutic. In fact, treatment with BCG is among the most effective cancer immunotherapies, and in high-risk, non-muscle-invasive bladder cancer, it is still the standard adjuvant treatment according to the European Association of Urology (EAU) guidelines (50, 51). BCG was used in seminal studies by Mackaness, who coined the term macrophage activation (classical activation) in the context of bacterial infection to describe the non-specifically enhanced, microbicidal activity of macrophages toward BCG (and Listeria) upon secondary pathogen exposure (52). The observation that vaccination with BCG also caused non-specific protective effects against non-related infections renewed the interest in this topic and led to the concept of “trained immunity” (53). At the cellular level, a first treatment with BCG resulted in enhanced responsiveness of monocytes and macrophages, which produced higher levels of cytokines and reactive oxygen species upon a secondary stimulation with BCG or even with non-related pathogens. A similar priming effect has been observed with β-glucan, a major component of the *C. albicans* cell wall (54). This increased responsiveness, which represents a form of innate memory, was a consequence of Akt–mTOR-driven metabolic reprogramming in macrophages and, importantly, increased flux through the mevalonate pathway appeared to be prerequisite for the establishment of trained immunity. The relevance of mevalonate metabolism was demonstrated when statins were shown to prevent the generation of trained immunity. This was consistent with the previous clinical observation that statin therapy has been associated with tumor progression leading to radical cystectomy in patients treated for bladder cancer with BCG (55). In addition, RNA sequencing combined with metabolomics revealed upregulation of multiple steps in the cholesterol synthetic pathway (54) (Figure 1).

Trained mevalonate metabolism leads to increased cholesterol biosynthesis, improving innate immunity. In addition, cholesterol is critically required for T cell growth and proliferation (6). T cell fitness has recently been demonstrated to specifically depend on high levels of free cholesterol in T cell membranes (56). Cholesterol esterification for storage purposes can therefore limit T cell activity (Figure 2B). Conversely, inhibition of the cholesterol esterification enzyme ACAT-1 was able to improve T cell responses and also improved the efficacy of immune checkpoint blockade by anti-CTLA-4 antibody in a mouse melanoma model (57). These findings collectively confirm the importance of mevalonate metabolism for cholesterol biosynthesis in antitumor immunity. Intriguingly, the efficacy of anti-CTLA-4 in mouse melanoma models depended on the microbiota of these mice (58), raising the important question of how the microbiota affects immunometabolism.

### Refraining from Undesirable Metabolism

Myc is obviously not only essential for tumor initiation *via* glycolysis-fueled mevalonate metabolism (30) but also for the maintenance of established tumors *via* FAO-driven OXPHOS (15, 59). For instance, triple-negative breast cancer displays a Myc-driven bioenergetic reliance on FAO (59). In addition, prostate tumors also exhibit low rates of glucose consumption and display increased OXPHOS driven by FAO (60). Particularly, prostate cancer, that becomes refractory to androgen deprivation therapy (61), critically depends on OXPHOS for growth and metastasis (62, 63). Likewise, leukemia (64) and glioblastoma (65) have been shown to require FAO for growth and survival. Consequently, inhibition of FAO has been suggested as a potential therapeutic strategy for this particular subset of breast cancer and possibly also for prostate cancer, glioblastoma, and leukemia.

The FAO inhibitor etomoxir targets carnitine palmitoyltransferase 1 (CPT1), which catalyzes the cytosolic formation of acyl carnitines at the outer mitochondrial membrane for mitochondrial import and subsequent oxidation of FAs (66). The etomoxir-mediated inhibition of FAO-driven OXPHOS decreases ATP levels and thus tumor cell viability and chemoresistance (64, 65). In addition, CPT1 inactivation in cancer cells resulted in increased sensitivity to oxygen and glucose deprivation as well as decreased tumorigenic potential *in vivo* (67). Unfortunately, however, clinical development of etomoxir has been discontinued because of severe liver toxicity. Currently, other inhibitors of CPT1 are clinically tested although not yet for their antitumor potential.

Although c-Abl-specific tyrosine kinase inhibitors (TKIs) substantially extend the survival of patients with chronic myeloid leukemia (CML), TKIs fail to eliminate leukemic stem cells resulting in minimal residual disease. Recently, primitive CML cells were shown to rely on upregulated OXPHOS for their survival, and intriguingly, combination treatment with the TKI imatinib and tigecycline, an antibiotic that inhibits mitochondrial protein translation, selectively eradicated leukemic stem cells both *in vitro* and in a xenotransplantation model of human CML (68).

As outlined above in the context of regulatory immune cells, additional engagement of FAO may divert acetyl-CoA from mevalonate metabolism (Figures 1 and 2A). The mevalonate pathway might thus become an Achilles’ heel of such tumor types and might therefore be targeted with statins, possibly as an adjuvant therapy preceding primary treatment. Importantly, statins may exhibit a dual effect in such a setting, since they can inhibit tumor growth or survival as well as hold downregulatory immune cells.

## Concluding Remarks

It is now becoming increasingly clear that mevalonate metabolism governs immune surveillance. However, cancer cells and in particular cancer stem cells may likewise depend on this metabolic pathway. Such a similarity in metabolic orientation between tumor cells and immune effector cells infiltrating the tumor microenvironment inevitably leads to a competition for the nutrients, metabolites, and oxygen that are required for fueling mevalonate metabolism and may ultimately even turn into a struggle for survival. While pharmacological inhibition of mevalonate metabolism in tumor cells may attenuate growth and proliferation, tonic flux through the mevalonate pathway in innate immune cells such as macrophages may contribute to trained immunity.

The additional engagement of FAO as it has been described for breast and prostate cancer cells may limit the availability of acetyl-CoA for mevalonate generation and metabolism. As a consequence, immune cells (T_reg_ cells and M2 macrophages) acquire regulatory function and tumor cells may undergo metastasis. Inhibition of FAO therefore appears to be desirable either as the primary therapeutic approach or as an adjuvant preceding cancer immunotherapy. In addition, the limitation of mevalonate pathway activity resulting from enhanced FAO might increase the sensitivity of tumor cells and regulatory immune cells to statins. Future personalized cancer medicine should include the assessment of the metabolic status of the patients’ tumor in order to develop the appropriate therapeutic strategies. Sequential therapy regimens might start with inhibitors of mevalonate metabolism and FAO to directly block tumor cells as well as regulatory immune cells, followed by immunotherapies that induce trained immunity in innate immune cells *via* mevalonate pathway stimulation.

## Author Contributions

All authors listed have made substantial contributions to text and figures and have approved the manuscript for submission.

## Conflict of Interest Statement

The authors declare that the research was conducted in the absence of any commercial or financial relationships that could be construed as a potential conflict of interest.

## Abbreviations

ABCA1, ATP-binding cassette transporter A1; ACAT-1, acetyl-CoA acetyltransferase 1; apo-AI, apolipoprotein A-I; ATP, adenosine triphosphate; ACL, ATP citrate lyase; BTIC, brain tumor-initiating cell; BTN, butyrophilin; CoA, coenzyme A; CPT1, carnitine palmitoyltransferase 1; CTLA-4, cytotoxic T lymphocyte-associated protein-4; DMAPP, dimethylallyl diphosphate; FA, fatty acid; FAO, fatty acid ß-oxidation; FAS, fatty acid synthesis; FPP, farnesyl diphosphate; GM-CSF, granulocyte/macrophage-colony-stimulating factor; GGPP, geranylgeranyl diphosphate; GPP, geranyl diphosphate; HMG, 3-hydroxy-3-methylglutaryl; HMGCR, HMG-CoA reductase; IFN, interferon; IL, interleukin; IPP, isopentenyl diphosphate; mTOR, mechanistic target of rapamycin; N-BP, nitrogen-containing bisphosphonate; OXPHOS, oxidative phosphorylation; PI3K, phosphoinositide 3-kinase; PP, diphosphate = pyrophosphate; SCAP, SREBP cleavage-activating protein; SREBP, sterol regulatory element-binding protein; TCA, tricarboxylic acid; TCR, T cell receptor; Th, T helper; TKI, tyrosine kinase inhibitor; TLR, toll-like receptor.
